# Cochlear Implantation Following Transcanal Infrapromontorial Approach for Vestibular Schwannoma: A Case Series

**DOI:** 10.3390/audiolres13010001

**Published:** 2022-12-21

**Authors:** Virginia Dallari, Enrico Apa, Daniele Monzani, Elisabetta Genovese, Daniele Marchioni, Davide Soloperto, Luca Sacchetto

**Affiliations:** 1 Section of Ear, Nose and Throat (ENT), Department of Surgical Sciences, Dentistry, Gynaecology and Paediatrics, Borgo Roma Hospital, University of Verona, 37134 Verona, Italy; 2 Department of Medical and Surgical Sciences for Children and Adults, Otorhinolaryngology Unit, Azienda Ospedaliero-Universitaria of Modena, University of Modena and Reggio Emilia, 41125 Modena, Italy

**Keywords:** inner ear, acoustic neuroma, skull base, simultaneous implantation, cochlear implant, normal contralateral hearing, tinnitus, case series

## Abstract

*Background*: Cochlear implantation (CI) following endoscopic transcanal infrapromontorial vestibular schwannoma (VS) dissection is a feasible intervention in intracanalicular VS, with minimal extension into the cerebellopontine angle, but no audiologic results have ever been reported in the literature. *Methods:* From 2015 to 2021 in the Otorhynolaryngology Departments of Modena and Verona, three patients underwent this intervention. All were suffering from sporadic left-sided intracanalicular Koos I VS. Intraoperative electrically evoked auditory brainstem responses and electrophysiological measurements were performed before and after the placement of the electrode array, respectively. Since device activation one month after the surgery, each patient was followed up with audiometric tests, data logging, electrode impedance measurements and neural response telemetry performed at each scheduled fitting session at 15 days and 3, 6, 12 and 24 months. *Results:* Only in patient No. 3, an auditory benefit was observed and still evident even 36 months after activation. Impedances increased progressively in patient No. 1 and a benefit was never reported. Patient No. 2 left the follow-up for worsening comorbidities. *Conclusions:* CI following transcanal infrapromontorial VS resection is a beneficial intervention. The residual cochlear nerve after the tumour dissection and the course of electrophysiological measurements in the postoperative period were the main predictive factors for audiological outcomes.

## 1. Introduction

Surgical procedures for vestibular schwannoma (VS) commonly involve three approaches: retrosigmoid, middle cranial fossa, and translabyrinthine approaches. The first two approaches have been used routinely over the last 50 years. These have the potential to preserve hearing, and their indications and effectiveness are well known [1]. In recent years, the introduction of endoscopic ear surgery has led to improved anatomical knowledge from the external auditory canal (EAC) to the internal auditory canal (IAC) [2]. This allowed the development of transcanal transpromontorial surgery, in which the EAC is used as a natural corridor to remove VS involving the IAC with or without minimal extension to the cerebellopontine angle (CPA).

In 2013, an exclusive endoscopic transcanal transpromontorial approach was proposed, which over the years was proven to be successful in the removal of small VS (stage I–II according to Koos classification), attributed to its better optics and direct visualisation of all the structures in the IAC and CPA [3,4]. After this encouraging experience, the transcanal corridor was used frequently, enlarging the surgical window to the CPA and extending the indications to VS of Koos stage II–III (expanded transcanal transpromontorial approach) [5,6,7,8]. Based on the low morbidity rate and good facial nerve preservation, this approach was described as very promising but not recommended if patients required a simultaneous cochlear implant (CI) placement, as proposed in the translabyrinthine approach. Marchioni and colleagues reported that in Koos stage I and II VS, complete removal of the cochlea is not necessary; rather, it is possible to partially preserve the basal turn of the cochlea and completely preserve the middle and apical turns [9]. The preservation of these anatomical structures, therefore, led to the description of the transcanal infrapromontorial approach, which allows fitting a CI to restore auditory function, especially in those patients in whom the function of the contralateral ear is hearing-impaired. This innovative approach is an excellent alternative for patients with small intracanalicular VS with limited extension (≤5 mm) into the CPA, where there is the intention to fit a CI on the same side [10].

The purpose of this study is to present our preliminary experience and to evaluate the audiologic results of the transcanal infrapromontorial approach for small VS with simultaneous placement of a CI.

## 2. Materials and Methods

The study was a retrospective multicentre case series. Three consecutive patients who underwent transcanal infrapromontorial surgery for intracanalicular VS (stage I–II according to Koos classification) and simultaneous CI placement were considered. Patients who underwent other approaches for VS removal or suffering from von Recklingausen disease or lateral skull base pathologies other than VS were not included in the study.

### 2.1. Interventions

**Figure 1:**
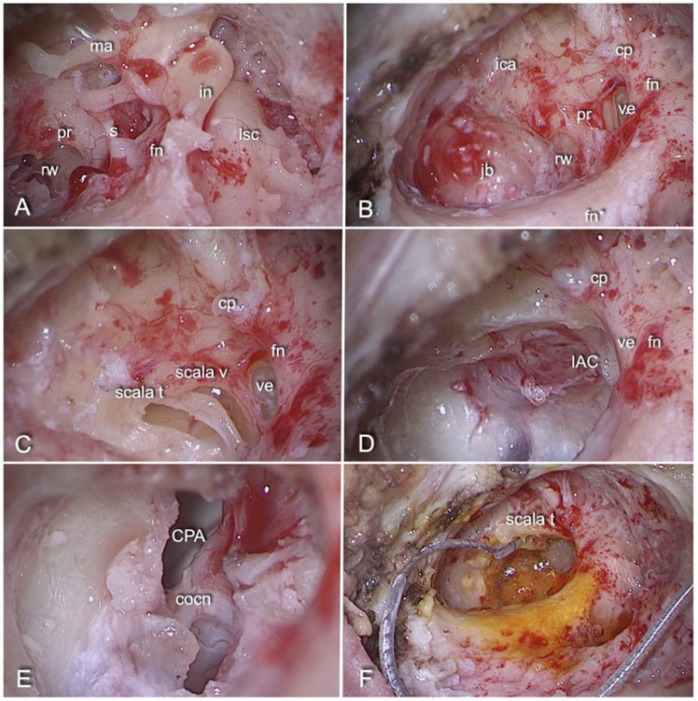
Step-by-step transcanal infrapromontorial approach with simultaneous cochlear implantation (microscopic view, left ear, patient No. 2). (**A**) Medial side of the tympanic cavity after retroauricular incision, EAC’s skin incision, drilling of the external auditory canal, removal of the tympanic membrane and chorda tympany sectioning (malleus *ma*, incus *in*, stapes *s*, tympanic portion of the facial nerve *fn*, promontory *pr*, round window *rw* and lateral semicircular canal *lsc*). (**B**) Main landmarks of transcanal infrapromontal dissection identifiable after a wide canaloplasty with removal of the ossicular chain (tympanic portion of the facial nerve *fn*, mastoid portion of the facial nerve fn*, vertical portion of the intrapetrous carotid artery *ica*, jugular bulb *jb* (cochleariform process *cp* and vestibule *ve*). (**C**) The scala tympani *scala t* and the scala vestibuli *scala v* identifiable after drilling with a diamond burr the tegmen of the round window and carefully opening part of the basal turn of the cochlea. (**D**) The cochlear-vestibular bone, the bony area between the cochlea, the vestibule and the fundus of the internal auditory canal *IAC*, created after removal of the most posterior and inferior portion of the cochlea basal turn. (**E**) The cochlear nerve *cocn* preserved after complete removal of the vestibular schwannoma (cerebellopontine angle *CPA*). (**F**) Placement of the cochlear implant array in the scala tympani through the opened basal turn of the cochlea.

The surgical treatment was performed under general anaesthesia by the same surgeon with proven experience in lateral skull base and cochlear implant surgery (D.Ma.) in the Otolaryngology Departments of Modena and Verona between 2015 and 2021. The same procedure was followed for each patient [11]. The patient was always positioned supine with the head slightly extended and rotated to the contralateral side. The surgeon mainly used a standard otologic microscope and, when necessary, a 0° endoscope 4 mm in diameter and 15 cm in length (Karl Storz, Tuttlingen, Germany). In addition, intraoperative facial monitoring was performed in each case. The main surgical steps are illustrated in Figure 1. Once the VS was completely removed, using the endoscope, the extent of tumour excision, the state of preservation of the nerve and contiguous vascular and nerve structures were inspected. In addition, the assessment of the functionality of the cochlear pathway was performed with the intraoperative electrically evoked auditory brainstem responses (eABRs) [12]. The test was considered positive if a clear wave V could be identified. After recording a clear V-wave in the eABRs, macroscopic anatomical preservation of the cochlear nerve was attempted without eABR hearing monitoring during tumour dissection. All patients received a state-of-the-art CI device by different manufacturers. After placement of the electrode array and before final closure, in order to confirm the correct positioning of the electrode array and the functioning of the cochlear implant, an experienced audiologist performed intraoperative electrophysiological measurements in the operating room using specific software provided by the CIs’ manufacturers. Impedance is the opposition to electrical flow [13]. Its measurements depend on the electrode–tissue interface, the fluid/tissue resistivity and the contact resistivity of the electrode and lead wires. Operating in stimulation and recording mode using two-way communication with the implant, it provides data on the integrity of the receiver/stimulator as well as the correct positioning of the array. Generally, the normal range of impedance is between 200 and 15,000 Ohm. Impedance anomalies such as short circuits (SCs) or open circuits (OCs) may be found [14]. The former are overly low values indicative of contact between two electrodes, whereas the latter are overly high values indicative of an open circuit resulting from the malfunction of an electrode or its placement outside the cochlea. In addition, the electrically evoked compound action potential (ECAP) measurement was performed [15]. This allows the measurement of electrically evoked compound action potentials in the auditory nerve and is recorded through the CI’s electrode array. It is used as a confirmatory measure for neural electrode connectivity and stimulation, confirming the proper position of the electrode array and the functioning of the device and assessing the possible cross-stimulation of the proximally found facial nerve.

Finally, adipose tissue harvested from the abdomen and covered by fibrin glue was used to close the defect between the inner and middle ear. The Eustachian tube was then closed with a temporalis muscle fragment and fibrin glue.

Patients were kept in a supine position for 48 h after surgery then gradually mobilised. A postoperative computed tomography scan (CT) was performed within six hours and repeated until five days after surgery in order to show the correct positioning of the array into the cochlea and exclude possible complications such as wound dehiscence with cerebrospinal fluid (CSF) leaking. In order to avoid septic and thromboembolic complications, prophylactic therapy with cefazolin and enoxaparin sodium was administered by default. After the hospital discharge, patients were evaluated in a specialist outpatient setting approximately 10–15 days after surgery. In case of no late complications and good healing of the retroauricular wound, CI was activated approximately one month after surgery. Finally, for each patient in whom the CI’s contribution to verbal perception was demonstrated, a speech therapy programme was planned for the first 3 months (a single 10-session course).

### 2.2. Follow-Up

Each patient underwent a comprehensive follow-up in specialised outpatient settings. A clinical assessment of facial nerve function was carried out at lateral skull base clinics with scheduled visits at hospital discharge and at three, six and twelve months after surgery. The House–Brackman grading classification (HB) was used in all cases, whereas the Sunnybrook grading system (SGS) was used in addition in the case of regional facial dysfunction [16]. During the same period, each patient was followed up in the audiology clinics with scheduled visits at 15 days at 3, 6, 12 and 24 months after CI activation. Clinical and audiological evaluations were performed. All audiometrical tests were performed in free field using a two-channel diagnostic audiometer (Otometrics MADSEN Astera2, Natus Medical, Taastrup, Denmark) and loudspeakers (Indiana Line, Valdagno, Italy). The patient was seated within a double-wall, soundproof booth that complies with ISO 8253 and in the visual field of the examiner. A warble tone was used as a sound stimulus because of its selectivity in the frequency range. In the case of contralateral normal or residual hearing, tests were performed using a set of headphones (TDH39P, Telephonics, Farmingdale, NY, USA) with masking narrowband noise (NBN) at 65 dB HL in the nonimplanted ear. At each fitting session, data logging and electrodes’ impedances were assessed, whereas the CI’s stimulation levels were adjusted according to ECAP, hearing outcome measures and the patient’s subjective judgment of loudness. Specific software provided by the CI manufacturers were employed.

### 2.3. Data Collection

Magnetic resonance imaging (MRI), data regarding past medical history, any concurrent treatments and vestibular and audiological assessments before the surgery were collected for each participant anonymously. Surgical videos and electrophysiological data recorded during the surgery were revised, and data regarding audiological follow-up and any specialist and rehabilitation assessments were collected as well. The three patients were assigned a random No. from 1 to 3 by the authors of this study (V.D. and E.A.).

This case series has been reported in line with the PROCESS Guideline [17].

## 3. Results

In the Otolaryngology Departments of Modena and Verona hospitals between 2015 and 2021, a total of 92 VS removals were performed using a transcanal approach: 53 transcanal transpromontorial, 32 expanded transcanal transpromontorial and 7 transcanal infrapromontorial approaches. Among those who underwent the last approach listed above, only in three cases (one in Modena and two in Verona) was it possible to preserve the cochlear nerve and therefore perform CI placement at the same time.

All were suffering from sporadic left-sided intracanalicular VS (Koos stage I) with a mean size of 12.33 mm (S.D. ± 0.58 mm). None had a facial dysfunction, and in all of them, the onset was characterised by progressive ipsilateral hearing loss coupled with tinnitus.

In patient No. 3, the symptoms occurred at the same time, whereas in patient No. 2, the tinnitus occurred two years before. In contrast, patient No. 1 initially presented with hearing loss followed by the onset of tinnitus and dizziness two and three years later, respectively. According to the Committee on Hearing and Equilibrium of the American Academy of Otolaryngology-Head and Neck Surgery (AAO-HNS), all participants presented a class C residual hearing function on the affected side [18]. On the normal side, hearing function was class B in patient No. 3 and class A in other patients. Due to the development of dizziness, in addition, patient No. 1 underwent otoneurological examinations with bithermal caloric tests according to Fitzgerald and Hallpike’s protocol. Parameters that were evaluated were the reduced vestibular response (RVR) and the directional preponderance (DP) using Jongkees’ formula and the peak of slow-phase velocity (SPV) [19]. Patients presented a slight bilateral hypofunction with peak SPV smaller on the left side. Other details are reported in Table 1.

**Figure 2:**
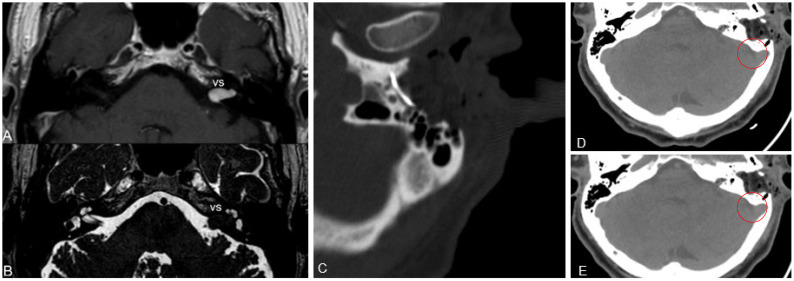
Preoperative and postoperative imaging of patient No. 1. (**A**) Axial gadolinium enhancement T1—weighted MRI. (**B**) T2—weighted drive HR MRI. (**C**) Postoperative CT—scan that shows the correct positioning of the array into the cochlea. (**D**) Suspected focal dilatation of the subarachnoid space (red circle). (**E**) Finding unchanged on follow-up CT scan (red circle).

All participants were candidates for surgery according to contrast-enhanced MRI (Figure 2A,B). In two cases, surgery followed a short wait-and-scan period and was due to evidence of rapid growth, while in patient No. 2, surgery was scheduled at the first documentation of the lesion. A review of the surgical videos showed that, in all cases, the cochlear nerve was anatomically preserved and the facial nerve was amenable to proper stimulation. Nevertheless, in patient No. 1, the vascularity of the surface of the cochlear nerve did not appear to be maintained; rather, the nerve appeared swollen and prone to bleeding. A clear wave V was identified in eABRs in all cases; hence, a state-of-the-art CI device was placed with the array gently positioned into the scala tympani. Patient No. 1 and patient No. 3 received a MED-EL^®^ Synchrony 2 Flex28 (MED-EL Medical Electronics, Innsbruck, Austria), while patient No. 2 received an Advanced Bionics^®^ HiRes Ultra 3D HiFocus Mid-Scala (Sonova Holding AG, Stäfa, Switzerland). Therefore, Maestro 9.0 and SoundWave Professional Fitting software were used to perform intraoperative electrophysiological measurements, respectively. For all patients, impedances were in the normal range, except for electrode 6 of patient No. 2, whose values were consistent with an OC. ECAP was observed in patient No. 1 from electrodes 1 to 8, in patient No. 2 on all electrodes tested and, finally, in patient No. 3 on 10 of the total 12 electrodes. On the postoperative CT scans, patients No. 2 and No. 3 presented with regular surgical findings. In patient No. 1, a hypodense areola was observed at the level of the superolateral side of the left cerebellar hemisphere with a density similar to CSF, consistent with a suspected focal dilatation of the subarachnoid space (Figure 2D). This finding was unchanged on the follow-up CT scan (Figure 2E). In all three patients, the postoperative CT scan confirmed correct electrode placement. Other postoperative details are reported in Table 2.

For all patients, CI activation was performed one month after surgery. None of them presented residual hearing function in the surgically treated ear. Impedances were stable for patient No. 1, decreased for patient No. 2 and increased for patient No. 3. ECAP was absent along the entire array in patient No. 2 and preserved on three and ten electrodes in patients No. 1 and No. 3, respectively.

In clinical terms, patient No. 1 complained only of residual dizziness, while for all patients, the level of facial dysfunction was improved. In detail, patient No. 1 presented a regional facial dysfunction of the marginalis nerve (I HB; 86/100 SGS), while patient No. 2 and patient No. 3 presented II and III HB facial dysfunction, respectively. In contrast, during the audiological follow-up, the course of the three participants was very different.

Patient No. 1 used the CI continuously for the first three months for about 7 h per day; however, the CI was never able to evoke real sound perceptions. Impedances gradually increased up to the first month and then remained stable, whereas ECAP was detectable but of low amplitude only on electrodes 4, 8 and 11.

Patient No. 2 discontinued the follow-up due to worsening comorbidities and never used CI continuously. At the fitting session six months after surgery, impedances were slightly increased, while ECAP was absent along the entire array.

**Figure 3:**
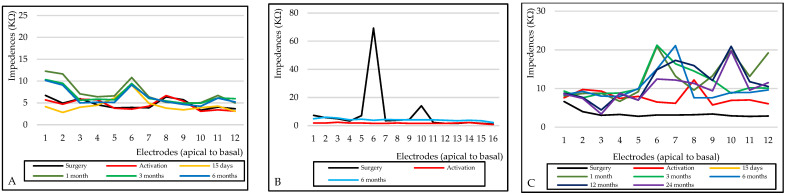
Electrodes’ impedance measurements during the follow-up. (**A**) Patient No. 1. (**B**) Patient No. 2. (**C**) Patient No. 3.

Patient No. 3 used the CI continuously for about 14 h per day. Impedances remained stable while ECAP was evident on 11 of the total 12 electrodes. At the audiometric evaluations, the tone threshold was present even if the threshold of verbal intelligibility was not reached (pure tone average at 500, 1000, 2000 and 4000 Hz around 45 dB and 50% signal recognition threshold reached at 30 dB; class B according to AAO-HNS). Impedances, after a slight increase, with values above the range only on some middle and basal electrodes, decreased and remained stable. Only for electrode 10, values compatible with OC were observed in the last fitting session, and it was, therefore, shut down out of caution. The impedance trend in the three participants is illustrated in Figure 3.

Patient No. 3 was the only one to complete the 24-month follow-up, while patient No. 1 and patient No. 2 were lost after six and nine months, respectively. At the last fitting session, 36 months after surgery, patient No. 3 presented a stable auditory and clinical situation with facial dysfunction III HB and residual hearing function class B, while impedances remained stable and ECAP was evident on all 11 active electrodes. At the last visit, patient No. 1 was stable with no facial dysfunction (I HB; 100/100 SGS) or dizziness. Currently, respective departments continue to follow patient No. 1 and patient No. 3 with periodical clinical and audiological visits.

## 4. Discussion

Several studies over the last ten years reported that cochlear implantation could safely be performed simultaneously with VS resection if the cochlear nerve was anatomically preserved and no overly aggressive manoeuvres were applied to remove the tumour. In addition to nerve preservation, audiological outcomes depended on tumour size in CPA and residual hearing function on the affected side [20,21]. Although a long-standing debate, an additional role may be played by a better contralateral hearing ear. In fact, good preoperative contralateral hearing status seemed to be a negative prognostic factor for CI performance on open-set discrimination, since a 28-fold higher risk of nonperformance with CI at 24-month follow-up was observed in the case of preoperative hearing classes A or B on the contralateral side of CI placement [22]. In contrast, in the case of normal contralateral hearing, satisfactory results were observed in terms of binaural benefits, such as sound localisation and improved speech perception, when the signal and noise came from different positions (squelch effect). Despite the slight improvement in tone and speech perception in these patients, the speech-in-noise perception and the subjective auditory benefit appear satisfactory [23]. Therefore, the good preintervention contralateral hearing function of the three participants should not be considered a limitation (Table 1).

Several approaches of surgery for VS were proposed, among which the transcanal infrapromontorial approach was proposed as an excellent alternative for patients with small intracanalicular VS with limited extension into the CPA. Given the removal of only the most posterior portion of the cochlear basal turn, CI could represent a feasible option [10]. In this study, a case series of three consecutive patients who underwent cochlear implantation after transcanal infrapromontorial resection of VS at the Otolaryngology units of Verona and Modena hospitals was reported. All were suffering from sporadic left intracanalicular Koos stage I VS, with a mean size of 12.33 mm (S.D. ± 0.58 mm). All received a state-of-the-art CI device, but the outcomes of the three participants were different, since only patient No. 3 demonstrated an auditory benefit. Intraoperatively, although the cochlear nerve appeared anatomically preserved and an eABR was present for all patients, patient No. 1 had a nerve that was swollen and prone to bleeding, with reduced vascularity on the surface of the cochlear nerve. In fact, visualisation of cochlear nerve continuity after tumour dissection is definitely the main factor in assessing whether to proceed with cochlear implantation [24]. Recently, it was observed that the integrity of nerve fibres along the entire course of the nerve, the whitish colour of the nerve after saline washing and the presence of vascularity on its surface could be predictors of a good outcome in more than half the patients undergoing CI after VS resection [25]. In addition to the aforementioned factors, involvement of the IAC fundus and tumour dissection technique (en bloc or piecemeal excision) have been observed as predictors of hearing outcomes [24].

Only patients No. 1 and No. 3 adequately and continuously used CI after activation, since patient No. 2 was not interested in using the CI and continuing follow-up due to worsening concomitant tumour disease, as well as no perceived benefit. Nevertheless, electrophysiological measures changed consensually throughout the course in all participants. A negative association between impedance and residual hearing was already reported in the literature. This could be due to a traumatic insertion of the array, an inflammatory response resulting in scar tissue formation and the influence of the array on the mechanical properties of the cochlea and residual hearing thresholds. Wimmer and colleagues observed higher impedances measured one month after surgery and their gradual decrement over time. Electrode contacts inserted more apically exhibited higher impedances, but basal electrodes maintained higher impedance for longer [26]. A similar trend was recorded in this study in patients No. 1 and No. 2 (Figure 3). In particular, two events are significant: the progressive increase in impedances in patient No. 1 and some values beyond the normal ranges for the basal electrodes in patient No. 3. The former was not uncommon in the postoperative period and already described as related to inner ear events leading to a loss of residual hearing and dizziness [26]. The second could be related to possible dilatation or scar tissue formation due to the removal of the posterior part of the cochlear basal turn. Finally, although its correlation with future implant performance remains uncertain, ECAP was absent since the activation session on the entire array for patient No. 2, present in three electrodes for patient No. 1 and present on 10–11 of the total 12 electrodes for patient No. 3, concurrently with the hearing outcome.

The involvement of a multidisciplinary team and following participants over quite a long period were the strengths of this study. In fact, while patients No. 1 and No. 2 experienced no benefit and were followed up until the device was used, patient No. 3 was followed up until 36 months after surgery. Otherwise, this case series presented several limitations, primarily the nonassessment of the self-perceived benefit of quality of life and auditory function. While its validation in the Italian language by our group is in press, a disease-specific index for quality-of-life assessment in VS patients exists and was already used [27]. A plethora of instruments assessing self-perceived speech perception, hearing disability and hearing quality are available. Moreover, although reliable tests assessing speech-in-noise perception as well as sound localisation and binaural cues were already used in clinical settings, assessment of these important aspects was omitted. Other considerable limitations were the lack of adequate evaluation of tinnitus and vestibular outcomes. The former appeared to have a considerable impact on VS patients, although a significant improvement in tinnitus after cochlear implantation was recently reported [28]. The latter is an issue often omitted in assessing such patients.

Finally, the small No. of patients may represent a methodological limitation, but it was consistent with the fact that this was a novel surgical technique indicated in limited cases, as demonstrated by there being only seven patients who underwent this procedure between 2015 and 2021 in the Otolaryngology units of Modena and Verona hospitals. Based on these considerations, after discussing VS patients in multidisciplinary tumour boards, when surgery is indicated, it should be performed in a high-volume centre and by an experienced surgeon because surgical experience influences outcomes [29].

## 5. Conclusions

Cochlear implantation following VS resection through the transcanal infrapromontorial approach is a reliable alternative in patients with intracanal Koos I stage VS, and good contralateral hearing does not represent a contraindication to operation. The decision whether to proceed with the implantation should be weighed considering the endoscopic features of the cochlear nerve such as the integrity of nerve fibres along its entire course, its whitish colour after saline washing and the presence of vascularity on its surface. Subsequently, telemetric electrophysiological measurements may represent a valid predictor of the outcome of auditory function during the follow-up. Their assessment is easy and quickly performed, does not require the active participation of the patient and is independent of middle ear status.

However, not much information is available regarding outcomes such as speech-in-noise perception, sound localisation, binaural cues, self-perceived quality of life, vestibular outcomes and the burden of tinnitus after the intervention.

Given the limited indications and its recent introduction to the surgical portfolio, cochlear implantation surgery following VS resection through the transcanal infrapromontorial approach should be reserved for high-volume centres and experienced surgeons.

## Figures and Tables

**Table 1:** Preintervention participants’ details.

	Patient No. 1	Patient No. 2	Patient No. 3
Gender	male	male	female
Age (years)	49	70	52
Body mass index	24	24.5	22
Comorbidities	diabetes mellitus type 1, autoimmune thyroiditis, autoimmune gastritis	prostate cancer, H. pylori gastritis	multiple breast adenomas, atrophic gastritis
Onset of symptoms ^1^	HL	T	HL and T
Hearing residual function ^2^	Class C	Class C	Class C
Contralateral hearing function	Class A	Class A	Class B
Symptoms at the time of surgery ^1^	HL, T and D	HL and T	HL and T
Facial dysfunction (HB)	I	I	I
Caloric test (r-SPV; l-SPV; DP) ^3^	11°/sec; 3°/s; 53% right	-	-
Previous management	wait-and-scan	-	wait-and-scan
Delay since onset of symptoms	three years	three years	six years
CI device	MED-EL^®^	AB^®^	MED-EL^®^
Site of surgery	Modena	Verona	Verona

^1^ HL = hearing loss; T = tinnitus; D = dizziness; ^2^ according to Committee on Hearing and Equilibrium of the American Academy of Otolaryngology-Head and Neck Surgery (AAO-HNS) [18]; ^3^ r-SPV = right peak of slow-phase velocity; l-SPV = left peak of slow-phase velocity; DP = directional preponderance [19].

**Table 2:** Postintervention participants’ details.

	Patient No. 1	Patient No. 2	Patient No. 3
Cerebrospinal fluid leaking	No	No	No
Facial dysfunction (HB)	II	II	III
Facial rehabilitation	No	Yes	Yes
Vestibular symptomatology	dizziness	No	No
Nystagmus	Right horizontal, grade I	Right horizontal, grade I	Right horizontal, grade I
Dizziness Handicap Inventory ^1^	14 (10; 0; 4)	-	-
Vestibular rehabilitation	Yes (a 10-session course)	No	No
Additional therapy	Prednisone ^2^, Choline Alfoscerate ^3^	-	-

^1^ Dizziness Handicap Inventory (physical, emotional, and functional domains); ^2^ 50 mg per day for 5 days, 25 mg per day for 5 days and 12.5 mg per day for 5 days; ^3^ 600 mg per day for 28 days.

## Data Availability

Raw data were generated at the Azienda Ospedaliero-Universitaria of Modena and at Borgo Roma Hospital of Verona. Derived data supporting the findings of this study are available from the corresponding author, D.S., on request.
